# Isolated limb perfusion for unresectable extremity cutaneous squamous cell carcinoma; an effective limb saving strategy

**DOI:** 10.1038/s41416-018-0149-z

**Published:** 2018-07-02

**Authors:** Eva A. Huis in ’t Veld, Dirk J. Grünhagen, Jan P. Deroose, Tamar E. C. Nijsten, Michel W. J. M. Wouters, Cornelis Verhoef, Winan J. van Houdt, Andrew J. Hayes

**Affiliations:** 1 Sarcoma and Skin Units, Department of Academic Surgery, Royal Marsden NHS Trust, London, UK; 2 Department of Surgical Oncology, Netherlands Cancer Institute, Amsterdam, The Netherlands; 3 Department of Surgical Oncology, Erasmus MC – Cancer Institute, Rotterdam, The Netherlands; 4 Department of Dermatology, Erasmus MC, Rotterdam, The Netherlands

**Keywords:** Squamous cell carcinoma, Surgical oncology

## Abstract

**Background:**

A small minority of patients present with locally advanced cutaneous Squamous Cell Carcinoma (cSCC). The aim of this study was to evaluate the effectiveness of Tumour necrosis factor α (TNF) and melphalan based isolated limb perfusion (TM-ILP) as a limb saving strategy for locally advanced extremity cSCC.

**Methods:**

A retrospective search from prospectively maintained databases, at two tertiary referral centers, was performed to identify patients treated with TM-ILP for locally advanced cSSC of an extremity between 2000 and 2015.

**Results:**

A total of 30 patients treated with TM-ILP for cSCC were identified, with a median age of 71 years (36–92) and 50% female. Response could not be evaluated in 3 patients. After a median follow up of 25 months, the overall response rate was 81% (*n* = 22), with 16 patients having a complete response (CR, 59%). A total of 7 patients developed local recurrence, with a median time to recurrence of 9 months (Interquartile Range 7–10). Progressive disease was observed in 5 patients (19%). Limb salvage rate was 80%. The overall 2-year survival was 67%.

**Conclusions:**

TM-ILP should be considered as an option in patients with locally advanced cSCC in specialised centers, resulting in a high limb salvage rate.

## Introduction

Non-melanoma skin cancer (NMSC) is the most frequent human malignancy^1,2^ with an increasing incidence reaching epidemic proportions among Caucasians in Europe, America and Australia.^3–8^ Approximately 80% of all NMSC are basal cell carcinomas (BCC) and 20% are cutaneous squamous cell carcinomas (cSCC).^6–9^

Usually cSCC presents as a localised lesion, for which various treatment strategies are available: wide surgical excision, Mohs surgery, cryosurgery, and radiation therapy. Margins of resection are an important prognostic factor for outcome^10^ and the primary aim of surgery is to remove the tumour in total with adequate margins. However some patients will present with locally advanced disease. In such locally advanced cases, a radical resection to achieve negative surgical margins may lead to serious loss of limb function, or may even require an amputation.

Regional chemotherapy by hyperthermic isolated limb perfusion was introduced as a therapy to treat advanced extremity malignancies either as a standalone treatment or as a neo adjuvant treatment to downsize a malignancy and facilitate function preserving surgery.^11,12^ Several reports show excellent response rates with tumour necrosis factor α (TNF) and melphalan based isolated limb perfusion (TM-ILP) for both melanomas with multiple in transit metastases and for locally advanced soft tissue sarcomas.^11,13–15^ Likewise, large series show that TM-ILP is a safe procedure with only a limited local toxicity without severe systemic toxicity.^16^ These observations led to the exploration of ILP for locally advanced cSCC of the extremities. This study aims to evaluate the effectiveness of TM-ILP as a limb saving strategy for locally advanced cSCCs in the extremities.

## Methods

### Patients

Between 2000 and 2015, all patients treated with TM-ILP for locally advanced cSCC were identified from a prospectively maintained database in one of the two tertiary referral hospitals: Erasmus MC—Cancer Institute, Rotterdam, the Netherlands, and Royal Marsden Hospital, London, United Kingdom. All patients were considered to be surgically treatable only by amputation or function disrupting ablative surgery because the extent of tumour affecting the limb. Unresectability of the tumour was based on the following factors: size, multifocality, number of recurrences, and tumour location (adherent to bones, blood vessels or nerves). All patients were discussed in a multidisciplinary tumour board. The majority of patients had chronic infected fungating tumours, and therefore almost all patients underwent TM-ILP with prophylactic systemic antibiotics.

### Perfusion

The ILP technique has been described extensively before.^17^ Briefly, after heparinisation, the surgeon isolated the targeted vessels from the systemic circulation and cannulated the vessels with silastic cannulae. The surgical approach for cannulation could either be at the inguinal, femoral, axillary, or brachial level depending on the location of the tumour. To prevent leakage a pneumatic tourniquet was used to compress collateral vessels. Leakage from the isolated circulation to the systemic circulation was measured continuously using technetium labelled albumin. A dose of 1 to 3 mg (arm) or 1 to 3 mg (leg) of recombinant TNF was injected as a bolus. The dose of melphalan raged between 50 and 80 mg for the perfusion of a leg and between 25 and 60 mg for an arm based on volume of the limb or body weight.^16^ After the perfusion, the limb was washed out.

### Toxicity

Acute regional toxicity after perfusion was classified according to Wieberdink et al.^18^ Regional toxicity and systemic complications were evaluated during the hospital stay or during follow up visits.

### Outcome

The primary outcome measure was response to TM-ILP. Response was measured by clinical examination and scored following World Health Organisation criteria.^19^ Time to local progression, systemic disease and overall survival were defined as time from ILP to the event.

The secondary outcome measure was limb salvage after TM-ILP. TM-ILP could potentially be a curative treatment or a neoadjuvant therapy to downsize a tumour prior to radical function preserving resection. Both of these approaches were considered to be limb salvage strategies.

### Statistical analysis

In this retrospective review, survival analyses were performed using the Kaplan Meier method. Differences between groups were assessed by the Log-rank test, T-tests and Fisher exact tests. A *p*-value of 0.05 was considered significant. Confounders for local progression and overall survival (OS) were identified using univariate analysis. Only confounders with a p-value below 0.1 were subsequently included in a multivariate Cox regression. Factors explored were: age, gender, hospital of treatment, site and size of the tumour (<5 cm, or >5 cm). IBM SPSS statistics 24 was used for the statistical analyses.

## Results

### Patients

A total of 30 patients were treated with TM-ILP of which 15 (50%) were female. Median age at time of TM-ILP was 71 (Interquartile Range [IR] 62–79). Median follow up was 25 months (IR 10–36). TM-ILP was offered in 18 patients with primary disease (60%), in 10 patients with local recurrent cSCC (33%), and two patients were known to have systemic disease and underwent a palliative ILP (7%). Patient, tumour, and treatment characteristics are summarised in Table 1.

**Table 1:** Patients, tumour and treatment characteristics

	*N* (%)	Median (range)
Gender		
Male	15 (50)	
Female	15 (50)	
Age		
In years		71 (36–92)
Size^a^		
≤5 cm	10 (38)	
>5 cm	16 (62)	
Site		
Arm	3 (10)	
Hand or wrist	5 (17)	
Leg	16 (53)	
Ankle or foot	7 (20)	
Number of tumours		
Unifocal	24 (80)	
Multifocal	6 (20)	
Disease stage cSCC at presentation		
Primary	18 (60)	
Recurrent	10 (33)	
Metastatic	2 (7)	
Concurrent metastasis		
None	25 (83)	
Lymph node	3 (10)	
Distant	2 (7)	
Surgical approach		
Axillary	3 (10)	
Brachial	6 (20)	
Femoral	20 (67)	
Iliac	1 (3)	
Doses		
TNF^b^ (mg)		2 (1–3)
Melphalan^b^ (mg)		60 (25–80)
Hospital stay		
In days		6 (1–72)

*cSCC*  cutaneous squamous cell carcinoma, *TM-ILP*  TNF and melphalan based isolated limb perfusion.

^a^unknown size (*n* = 4).

^b^median dose in mg

### Treatment outcome

Response could be evaluated in 27 patients. In three patients response could not be evaluated because of, amputation (*n* = 1), or death (*n* = 2) within 3 months after ILP.

**Fig. 1:**
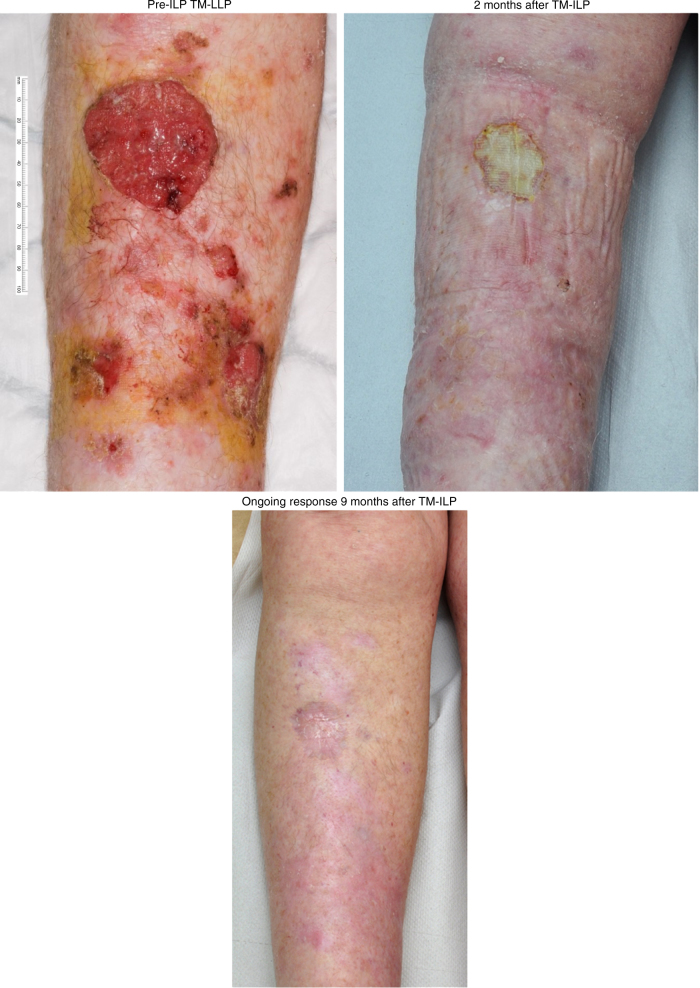
Course of a complete response of the lower leg. Ongoing response 9 months after TM-ILP. *TM-ILP* TNF and melphalan-based isolated limb perfusion

The overall response (OR) rate of these 27 patients was 81% (*n* = 22). Sixteen patients had a complete response (CR, 59%) and six patients had a partial response (PR, 22%). An example of a complete response is depicted in Fig. 1. Five patients (19%) had progressive disease (PD) within 3 months after TM-ILP. No differences in tumour size (*p* = 1000), stage of disease (*p* = 0.248), number of tumours (*p* = 0.246) and tumour location (*p* = 0.263) were found between the different response groups (Table 2).

**Table 2:** Patient characteristics per response to ILP

	CR (*n*, %)	PR (*n*, %)	PD (*n*, %)	*p*-value
Size				1.000^F^
<5 cm	6 (43)	2 (50)	2 (40)	
>5 cm	8 (57)	2 (50)	3 (60)	
Number of tumours				0.246^F^
Unifocal	13 (81)	5 (83)	3 (60)	
Multifocal	3 (19)	1 (17)	2 (40)	
Disease stage cSCC at presentation				0.248^F^
Primary	10 (63)	4 (67)	2 (40)	
Recurrence	2 (12)	1 (17)	1 (20)	
Multiple recurrence	4 (25)	1 (17)	2 (40)	
Location				0.263^F^
Arm	2(13)	0 (0)	1 (20)	
Wrist or hand	2 (13)	2 (33)	0 (0)	
Leg	7 (44)	3 (50)	4 (80)	
Foot or ankle	5 (31)	1 (17)	0 (0)	

*F* Fisher exact test, *CR* Complete response, *PR* Partial response, *PD* progressive disease, *cSCC* cutaneous Squamous cell carcinoma

### Surgical intervention

Of all 30 patients, at least 19 patients were candidates for amputation before ILP. Of these patients, 13 patients did not need surgical intervention after ILP (68%), 1 patient needed resection (5%) and 5 needed amputations (26%).

**Fig. 2:**
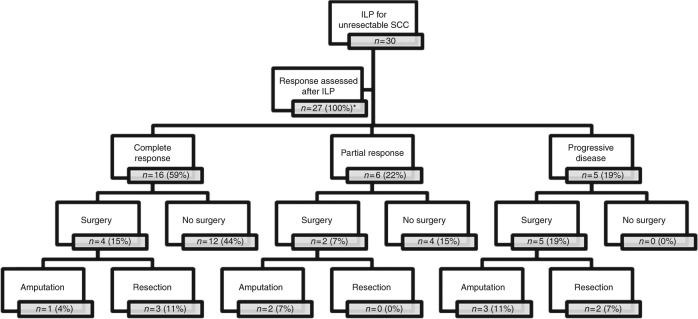
Consort diagram of patients per response group and surgical intervention. *In three patients response could not be evaluated

Analysing all 30 patients, 12 patients eventually needed surgical intervention after TM-ILP (40%), which consisted of resection of tumour in 5 patients (17%), and amputation in 6 patients (20%)(Fig. 2). One patient (3%) needed surgical intervention but refused.

Of the five patients who underwent a limb conserving resection of residual or remaining tumour, two patients underwent resection of residual tumour and the tumour was found to be 100% necrotic on histopathological analysis. Both of these patients were classified as CR. Two patients had PD with already metastatic disease at time of ILP. For these patients ILP was done with palliative intent because of intractable pain and/or uncontrollable wounds, but due to progression surgery was unavoidable. One patient had a clinical PR and underwent a surgical resection of residual tumour which demonstrated viable tumour on histopathological analysis.

Of the 6 patients who underwent an amputation after TM-ILP, one patient had an initial CR but subsequently needed amputation after development of local recurrence. After resection of the recurrence this patients suffered from severe wound healing problems and amputation was unavoidable. Three patients had an amputation because of PD, and one patient had a PR but still needed amputation. Finally, one patient needed amputation due to ischemia. The median time between TM-ILP and amputation in these 6 patients was 3 months (range 0–9).

### Local progression

Of the 27 patients were response could be evaluated, five patients (19%) showed no response to TM-ILP and had immediate PD. Of all 30 patients, another 7 patients (23%) developed local progressive disease during follow up. The median time to local progression for these 7 patients was 9 months (IR 7–10) (Table 3). The local progression free survival (PFS) was significantly longer in the CR group, compared to the PR group (Log-rank, p = 0.018), whereby the median PFS for both groups was not reached. None of the following factors were associated with duration of PFS: age (*p* = 0.216), gender (*p* = 0.560), hospital (*p* = 0.187), site (*p* = 0.862), and size of the tumour (<5 cm, or >5 cm, *p* = 0.939).

**Table 3:** Response, local recurrence and systematic disease

Type of response^a^	Patients (*n*, %)	Local progression (*n*, %)	Months to local progression (median, range)	Metastatic disease^b^ (*n*, %)	Months to metastatic disease (median, range)
Complete response	16 (59)	4 (25)	9 (9–22)	4^c^ (25)	0 (0–21)
Partial response	6 (22)	3 (50)	4 (2–6)	3 (33)	3 (3–9)
Progressive disease	5 (19)	N/A	0 (0–0)	3^d^ (60)	0 (0–4)
Overall response	22 (81)	7 (23)	9 (2–22)	10 (32)	3 (0–21)

^a^for 3 patients response could not be assessed.

^b^metastatic disease includes regional and distant metastasis.

^c^3 patients already had metastatic disease at time of ILP.

^d^2 patients already had metastatic disease at time of ILP

### Systemic metastasis

A total of 3 patients in this cohort had lymph node metastases at time of ILP (10%) and were treated with lymph node dissection concurrent with ILP. In addition, 3 more patients (10%) developed lymph node metastases in the follow up and were treated with lymph node dissection with a median time to lymph node metastases of 4 months (range, 0–9) (Table 3). Systemic metastases were present in two patients (7%) at time of ILP, while two more patients (7%) developed systemic disease during follow up: 3 and 21 months after ILP (Table 3).

### Overall survival

Eighteen patients were alive at last follow up (60%) with a median follow up for this particular group of 30 months (IR 18–38). In 14 patients (47%) there was no evidence of disease at last follow-up. Twelve patients (40%) died during follow up, of whom 6 (20%) died of advanced disease, 2 (7%) of complications after surgery, and 4 (13%) of reasons not related to cSCC. Median OS was 54 months (95% CI: 20.4–87.6), with a 2 year survival of 67%.

Gender (*p* = 0.187), hospital (*p* = 0.748), site of cSCC (*p* = 0.241), and size of the tumour (<5 cm, or >5 cm, *p* = 0.370) did not influence overall survival.

### Complications

The vast majority of patients in this series did not experience serious local toxicity. In 25 patients (83%) Wieberdink I or II was observed and in four patients (13%) Wieberdink III. One patient (3%) underwent an amputation 9 days following TM-ILP (Wieberdink V) due to ischemia. Leakage to the systemic circulation was minimal in all procedures, in only one case a leakage of 5% was noted, while in all other cases leakage was 2% or lower. The 30 mortality following TM-ILP for advanced cSSC was 0, however, two patients died 66 and 71 days after perfusion. The first patient was admitted to the Intensive Care Unit with respiratory distress after his operation and eventually died of pneumonia and respiratory failure. This patient had severe comorbidity, including severe chronic pulmonary obstructive disease and pre-existing diabetic complications (renal failure and hypertension). The procedure of the second patient was complicated due to a rupture of the brachial artery that was cannulated for TM-ILP. The patient was re-operated in an outside hospital and a vascular prosthesis was inserted. The patient died two months after TM-ILP of septic complications caused by an infected vascular prosthesis.

## Discussion

This unique multicentre experience of 30 TM-ILPs as treatment for locally advanced cSCC and demonstrates that TM-ILP is a valuable limb saving strategy in selected patients who would otherwise need an amputation or function disrupting ablative surgery. To our knowledge, this is the largest report on the outcome of ILP for locally advanced cSCC. With an overall response rate of 81%, a CR rate of 59%, and a limb salvage rate of 80%, this data shows that ILP is an effective treatment to obtain local control for locally advanced cutaneous squamous skin carcinoma. Almost half of the patients were free of disease at the end of follow up (*n* = 14, 47%).

Reports in the literature of oncological outcomes for advanced unresectable cSCC treated by ILP or other modalities are rare. One small study reports a series of 12 ILPs treated at multiple centres for cSCC with comparable results to this study (CR of 67% and 75% limb salvage rate).^20^

Other treatment modalities described for advanced cSCC at any site include conventional cytotoxic chemotherapy, newer systemic therapies, radiotherapy, and combinations of these modalities.^21–30^ The are a number of small case series of patients with advanced cSCC treated with cytotoxic chemotherapy usually involving cisplatin.^22,24,25^ Only 2 studies included more than 10 patients, one reporting a CR in 4 out of 14 patients using a Cisplatin, 5-FU and bleomycin regime^22^, and another reporting a PR rates of 56% and 47% using either platinum or taxane based chemotherapies respectively. Immunotherapy is mostly described using interferon alpha with or without chemotherapy, with the highest CR of 50% when combining Retinoid acid, interferon alpha and Cisplatin.^23–26^

More recently targeted therapies have been uses for advanceds cSCC.^25,28–30^ Two large studies evaluated the effectiveness of cetuximab and panitumumab in locally advanced and metastasised cSCC.^28,30^ Maubec et al. found a CR of 6%, a PR of 26% and an overall survival 8.1 months with cetuximab, with 47% of the patients having locally advanced cSCC.^28^ Foote et al. found an OR of 31% with panitumumab, including the 13 patients with locally advanced cSCC.^30^

There is very limited data regarding durable responses of radiotherapy as single treatment in locally advanced cSCC, however adjuvant radiotherapy can significantly improve survival in patients with lymph node metastases.^23^

Although the side effects of the TM-ILPs were low in this cohort, we did observe two (7%) in hospital deaths within 3 months after TM-ILP which is rare for ILP treatment. One of these patients died of pulmonary complications, while this patient was known to have severe co-morbidity including diabetes and severe COPD. Given the low leakage rate it is unlikely that his postoperative respiratory problems were caused or exacerbated by systemic leakage of the drugs, although we cannot rule this out completely. The second patient died of a complication that was directly attributable to ILP (sepsis subsequent to an infected arterial graft after an rupture of the brachial artery at the site of cannulation). Experience from larger series of ILP for the treatment of sarcoma and melanoma indicate that the mortality rate associated with ILP is less than 1%^15–17,31–34^ Of interest, both patients were classified as American Society of Anesthesiologists physical status classification  (ASA)—three patients, and both patients were treated in the early stages of this ILP treatment. Both centres are far more experienced with the procedure now than 15 years ago, and patient selection is more strict when it comes to patients with (severe) co-morbidity.

This study is limited by the relatively small number of patients, and therefore the results should be read with caution. Also, outcome could not be assessed in 3 out of 30 patients potentially leading to a bias in response rates. Nevertheless even with these caveats the response rates remain higher than other reported treatments.^21–30^

Of interest, the agents used for ILP are derived from the melanoma ILP protocol. In literature, results of ILP are reported combining doxorubicin with TNF-a showing similar results.^35,36^ These results are promising, and exploration of other chemotherapeutic agents to possibly achieve even higher response rates could be interesting for the future.

In conclusion, TM-ILP is an effective treatment option for patients with locally advanced cSCC based on this study. Altogether, TM-ILP should be considered as an option in locally advanced cSCC patients.

## Availability of data and materials

All data used for this research is de-identified and stored in SPSS files or Excel spreadsheets.
